# Book Notices

**Published:** 1870-11

**Authors:** 


### Book Notices.

A Treatise on the Theory and Practice of Obstetrics. By W. H. Byford, A.M., M.D., Prof. of Obstetrics and Diseases of Women and Children in the Chicago Medical College, etc., etc., author of the Practice of Medicine and Surgery applied to the Diseases and Accidents incident to Women; Chronic Inflammation of the Unimpregnated Uterus, etc., etc. New York: Wm. Wood & Co., 61 Walker Street. 1870.

This is an octavo volume of about 460 pages, published in good style, and amply illustrated by cuts. It is a concise, plain, practical treatise on midwifery, convenient, both as a text-book for students, and a work of reference for the practitioner. The subjects treated are arranged under the following heads: The Pelvis—the Fœtal Head—Female Organs of Generation—Generation—Displacements of the Uterus during Pregnancy—Abortion and Miscarriage—Labor and its Varieties—Anæsthetics in Labor—Natural Labor in Multiple Cases—Attentions to the Child—Puerperal Condition—Difficult Labors—Hemorrhage after Expulsion of the Fœtus—Inversion of the Uterus Complicating Labor—Rupture of the Uterus—Prolapse of the Cord—Puerperal Convulsions. We freely commend the work to the patronage of the profession.

For sale by W. B. Keen & Cooke, and booksellers generally.

Transactions of the Twenty-fifth Annual Meeting of the Ohio State Medical Society, held at Cleveland, June 14–16, 1870.

This is a neatly published and bound volume of 300 pages; containing the following: Minutes of the Twenty-fifth Annual

Meeting; Address of the retiring President; Surgical Applications of Carbolic Acid, by P. S. Connor, M.D., Cincinnati, O.; Climatology and Diseases of Southeast Kansas, by P. Beeman, M.D., Iola, Kansas; Report of Committee on Anatomy Law; Historical Review of Medical Organizations in Ohio, by Edward B. Stevens, M.D., of Cincinnati, O.; Report on Hæmatics, by E. H. Wyatt, M.D., Delaware, O.; Report on Vaccination, by W. B. Davis, M.D., Cincinnati, O.; Report on Pneumonia, by O. G. Selden, M.D., Shanesville, O.; Prevailing Diseases, by J. B. Black, M.D., Newark, O.; Temperature of Nervous Diseases, by W. G. Conklin, M.D., Dayton, O.; Cantharisom, by Alex. McBride, M.D., Berea, O.; Report on Obstetrics, by Edward B. Stevens, M.D., Cincinnati, O.; A Poem, by John M. Brown, M.D.; Constitution of the Ohio State Medical Society, and Code of Ethics of the American Medical Association; List of Members.

Many of the foregoing papers are worthy of a careful reading, and the volume is highly creditable to the Society.

